# The status quo of research in sustainable FDI: exploring the theoretical agenda and policy inferences in West and Central Africa

**DOI:** 10.1186/s43093-022-00153-5

**Published:** 2022-10-01

**Authors:** Jacques Yana Mbena

**Affiliations:** 1 Monarch Business School, Zug, Switzerland; 2 University of Sorbonne Paris Nord, Villetaneuse, France

**Keywords:** Sustainable FDI, West and Central Africa, "African sustainable growth tragedy", Policy implications

## Abstract

**Purpose:**

This paper investigates the status quo in the literature on sustainable foreign direct investment "FDI" in West and Central Africa. It is believed that utilizing the FDI Qualities Policy Toolkit developed by the Organisation for Economic Co-operation and Development "OECD" will help identify which policies are acknowledged today and the ones that may need particular attention from academia and policymakers.

**Design/methodology/approach:**

The paper utilizes a literature triangulation of FDI, sustainability, and economic development theories to extract the seed of a narrative helping to capture the current theoretical agenda and policy implications around the phenomenon of sustainable FDI in West and Central Africa. We selected and scrutinized (*N* = 53) articles published in various academic journals between January 2019 and March 2022 and investigating issues around sustainable FDI. The OECD framework later assisted in mapping today's theoretical agenda and policy inferences related to sustainable FDI in the region.

**Findings:**

It is acknowledged that there is an ongoing theoretical discussion informing on policy implications around the subject of sustainable FDI in West and Central Africa. It seems to be a consensus about the role of FDI's quality in enhancing sustainable regional growth. However, the use of the taxonomy has shown a clear focus on macroeconomic and ecological determinants reinforced through the OECD-defined policy dimensions of technical and financial support and international agreements and standards. The review allowed the perception of a theoretical gap in sustainability outcomes around the subjects of social justice in general and gender diversity in particular. It is recommended that academia and policymakers emphasize FDI theories and policies around the OECD framework of governance and domestic regulations.

**Originality/value:**

The paper informs through a literature-based review on determinants that academia and policymakers need to give particular attention to for better addressing all subjects around sustainable FDI in West and Central Africa. The paper proactively advises on specific phenomena that should be considered to avoid the fulfillment of the hypothesis of an "African sustainable growth tragedy."

**Supplementary Information:**

The online version contains supplementary material available at 10.1186/s43093-022-00153-5.

## Introduction

Financial decisions are classified as FDI when organizations or private persons acquire company's business assets headquartered in a foreign country. Despite the economic meltdown resulting from the COVID-19 pandemic, FDI flows hit $1 trillion in 2020 [46]. The United Nations Conference on Trade and Development "UNCTAD" ([46], pp. 248–251) reported that, compared to other regions (e.g., Europe), the decrease in FDI flows in Africa was less significant. However, as the net flow that the continent benefited from reached approx. $39 billion compared to $87 billion for Latin America and the Caribbean or $72 billion for Europe, Africa remained at the bottom of FDI inflows in 2020. With respect to the findings of Borensztein et al. [11], it is believed that despite the above-reported decreased and low level of FDI flows, foreign capital continues to play an undeniable role in the continent economic growth and development.

### Reviewing research on FDI in West and Central Africa

FDI has always been used as a vector for enhancing economic growth in Africa. Fafchamps [20] noted that despite a decade of economic growth, actionism, policy implications, and applied strategies may not have been as determinant as expected. Avom and Ongo Nkoa [37] later tried to capture the impacts of FDI on Central African economic growth. Yusuf, Shittu, Akanbi, Umar, and Abdulrahman [49] explored the role that FDI, governance, and political stability play in Western African growth. Ongo Nkoa and Song [38] argued that appropriate macroeconomic measures and development strategies contributed to and continue to strengthen the rise of the FDI ratio in terms of gross domestic product "GDP" in the region. However, the observed declining and relatively lower FDI ratio to GDP in West and Central Africa compared to other African sub-regions lead to questioning the sustainability of current settings.

Several studies have investigated FDI in general [3, 23]. The diversity of FDI theoretical streams has not simplified any attempt to find a consensus on how to aggregate them. Kindleberger [26] presumed that most people would agree with the argument that if perfect markets were given, FDIs would no longer exist. Following this point of view, Denisia [16] argued that FDI theories could be categorized into either perfect or imperfect market theories. Nayak and Choudhury [32] extended FDI theories into six categories based on macroeconomic arguments. It is presumed that other FDI dimensions, such as social justice or sustainability, are not equally explored within the literature.

Scholars are already going beyond traditional approaches to address specific regional challenges. For example, while Morrisset [29] and Fofana [21] investigated diverse public policy implications, Moss et al. [30] acknowledged the influence that the dimensions of trust and perceptions may play in nurturing FDI in Africa. However, it is observed that academics and policymakers around the continent focused only on how to foster FDI in volume. Hence, they may have missed looking at qualitative elements equally. This observed deficiency may somewhat explain regional economies' lack of resilience in the event of economic meltdown. The hope is that challenges resulting from pandemics such as COVID-19 may accelerate the consideration of social responsibility and sustainability perspectives in West and Central African FDI theories and policy.

Interestingly, it is informed that FDI flows in West and Central Africa are mainly directed to business opportunities within the primary sector [45]. Researchers argue that such investments may have long-term mitigated results. The findings of Tornell and Lane [43] about the "voracity effect" contend that natural resources may negatively impact the growth of fragile countries. Torvik [44] argues that a natural resources-abundant economy may experience inverted macroeconomic effects due to the lack of diversification, especially in the industrial sector. Remarkably, Cecchetti and Kharroubi [12] presume that financial development related activities enhance human capital skilling and economic growth. It is understood that besides how knowledge and technologies are transferred, the industry in which FDI flows are directed also impacts FDI outcomes.

The World Bank questioned if the observed investors' focus on the natural resources-rich primary sectors may have contributed to a less diversified economic ecosystem in West and Central Africa [48]. Considering the impact that global phenomena such as the pandemic COVID19 have on the resilience capabilities of African economies, the assumptions of Easterly and Levine [18] about the hypothesis of an "African growth tragedy" are believed to regain importance. It is presumed that next to the volume of FDI flows, their quality or sustainability attributes may have determining effects on sustainable economic growth and development.

### Sustainable growth and FDI instruments

Academics argue that knowledge and technology transfer that follow multinationals' investment abroad positively impacts host countries' economic growth [47]. According to Borensztein et al. [11], when a country benefiting from FDI flows has a significant stock of human capital, FDI may contribute to economic growth and development. In their works, Borensztein et al. [11] argue that: "The beneficial effects on growth of FDI come through higher efficiency rather than simply from higher capital accumulation" (1998, p. 6). This conclusion partly supports the hypothesis of FDI's quality relevance.

Scholars have not agreed on a definition of what sustainable FDI is all about. The OECD [35] enumerated the main components of sustainable FDI: (1) economic development, (2) good governance, (3) social development, and (4) environmental sustainability. It is believed that sustainable FDI can be defined as FDI that produces sufficient returns for maintaining business continuity and engagement without hurting the interests and long-term development goals of the beneficiary country.

Despite the rise of sustainable fund investments from $1.3 trillion to $ 3.9 trillion between 2010 and 2020, the UNCTAD [45] reports that only 96 billion sustainable funds were invested out of developed countries in 2020. Furthermore, the estimated volume of sustainability-dedicated investments or investments in products targeting sustainable development products was approximately $3.2 trillion in 2020 [45]. Interestingly, the market for sustainable investments is regionally concentrated. This market consisted by 2020 of $1.7 trillion in sustainable funds, $1 trillion in green bonds, $212 billion in social bonds, and $218 billion in mixed-sustainability bonds [45]. The UNCTAD observed that most flows in sustainable investments are: "…domiciled in developed countries and target assets in the developed markets…questions remain about greenwashing and its impact on sustainable development. Nevertheless, the sustainable investment market's rapid expansion indicates the potential for capital markets to help fill the financing gap to attain the sustainable development goals (SDGs)" [45], p. 208. Next to the existing academic discussion about the readiness of African institutions to attract and foster sustainable economic growth [4, 6], it may be relevant to question how Africa and other less developed regions can have better access to sustainable projects financing.

Like developed countries, most low- and middle-income countries faced severe socio-cultural and economic challenges and experienced negative growth due to the pandemic. However, while the first one continues to get easily financed, the last one is struggling to get the necessary financial support for their respective economies. The International Monetary Fund "IMF" stated that developing countries required $2.5 trillion to meet their pandemic-related financial needs [35]. It is observed that the crisis of COVID-19, a pandemic that rapidly turned into a worldwide economic meltdown, provoked a new debate about the ability to develop countries to reach the defined Sustainable Development Goals "SDGs" post-COVID-19.

Similar to past crises, the pandemic COVID19 seems to confirm the dependency of developing economies on FDI. It can be questioned why the cumulated flows of FDI that these countries benefited from for decades have not so far contributed to more resilience and sustainable economic growth, which in turn may have moderated the impacts of global crises like COVID19. The OECD argues that: "FDI can play a crucial role in making progress toward the SDGs by advancing decarbonization, increasing innovation, creating quality jobs, developing human capital, promoting gender equality and raising living standards." [36], p. 6. For building sustainable and resilient economic growth and development, the quality of FDI flow is assumed to be as significant as its volume. The paper explores the ecosystem of measures resulting from policy-driven approaches to enhancing sustainable FDI in West and Central Africa and attempts to investigate whether the view of the OECD that policies and institutional arrangements play a critical role in maximizing investments outcomes [36], p. 6, is acknowledged and shared within the literature. The paper questions FDI ambiguity and paradigms for sustainability policies in the region and utilize a systematic literature review to examine: Which qualitative and specific sustainability dimensions of FDI lawmakers and academia should take into consideration for nurturing sustainable economic growth and development in West and Central Africa.

## Related articles

The literature review process informs the theoretical agenda right before and during the pandemic of COVID19 on (1) FDI academic literature in Central and West Africa; (2) policymaking analysis; and (3) sustainability characteristics in both policy and academic literature from January 2019 till March 2022.^1^The article built upon Newman and Gough's [33] literature review findings and observed best practice [27] to support the methodological validation and repeatability of the literature reviews: The seven steps of critical literature review proposed by Juntunen and Lehenkari [24] were implemented: (1) selecting research questions, (2) selecting bibliographic or paper databases; (3) selecting search criteria; (4) applying practical screening criteria; (5) applying methodological screening criteria; (6) conducting the review; and (7) synthesizing the results (Additional file 1).

### Review scope, journals, articles, and reports selection

With the research question already defined above, the paper followed step 2 of the literature review process by screening annual reports of international organizations and academic journal publications. The regional focus of the paper implied a high likelihood of a low number of available publications within specialized journals [25, 28]. Therefore, it was presumed that pre-selecting specific journals might have resulted in insignificant data collection. In this first phase of this step, the framework developed by the OECD for policies on the impact of investments on sustainable development [36], p. 6 was identified. It was assumed that this framework could be used for screening policy and implications in sustainable FDI research within West and Central Africa. In the next phase, the following keywords were used: foreign, direct, investment, and Africa within the Web of Science Core Collection to identify, regardless of their sources, relevant publications issued from January 2019 through March 2022. This step resulted in 584 publications. Table 1 summarizes further applied steps, search filters, and specific rationales, and “Appendix 1” informs on the methodical replicability and screening procedures.

**Table 1:** Search filters and results

Search map	Keywords	Focus area	Other results/rationales	No. of publications
Step 2: Basic scan of the Web of Science Core Collection	Foreign, direct investment, and Africa	–	–	584
Step 3: Calibration	Central Africa, West Africa, Covid, policy, sustainability, sustainable, technology transfer, knowledge transfer, emission, social responsibility and justice	Sustainability and policy	Language English; only articles	93
Steps 4 and 5: Investigation of the title, abstract and text	Sustainability and policy	West and Central Africa	Exclusion of conceptual works and narrative literature reviews	53
Grouping 1: "Sustain"	Sustainability, sustaining, renewable energy, emission, environment, and pollution	West and Central Africa	–	32
Grouping 2: "Others"	–	West and Central Africa	Remaining articles after Grouping 1	21

The paper advanced the methodological screening by categorizing the articles based on their titles or abstracts with the tag "obvious" and "applied or inferred." It is believed that the clearer a title or an abstract is, the more likely the article informs on specific outcomes or implications on sustainable FDI policies.

### Articles and journals with specific indications

#### Articles indicating FDI policies and implications

Some articles included policy and governance-related terminology or the results of past policing within their title. For example, the conclusions of Shittu et al. [41] inform that: "…the authors found that FDI stimulates the growth of the sub-region, while political governance enhances the positive impact of FDI on economic growth" [41], p. 1733. A total of (*N* = 12) articles was observed within that category.

#### Articles with "applied or inferring" policy implications

Some articles (*N* = 9) were tagged as inferred or applied as their findings informed on broader perspectives or sectorial macroeconomic implications and FDI’s consequences on economic growth and development. For example, the work of Osabohien et al. [39] may not have the word policy in its title but stresses the importance of policies for inclusive growth. The authors defend that: "Therefore, the study recommends that effective policies such as flexible trade policies to enhance the exchange of goods and services should be implemented, which is crucial given the need for more resilience in post-COVID-19 ECOWAS" [39], p. 1. It is understood that the article inferred the importance of policies in international business relations for inclusive growth.

#### Articles with policy indications in sustainability-oriented publications

The work of Duodu et al. [17], titled: "Foreign direct investments and environmental quality in sub-Saharan Africa: the merits of policy and institutions for environmental sustainability," provides a clear policy indication. The authors also strongly argue for a more sustainable FDI policy: "…The study concludes that policies and institutions for environmental sustainability in SSA are important as they improve environmental quality. The study also finds policies and institutions for environmental sustainability complement FDI to improve environmental quality in the long run [17], p. 66101. Only (*N* = 3) similar articles were marked as obvious for including the word policy or reforms in their title and informing on sustainability characteristics.

#### Applied policy indication versus inferring policy indications

The articles grouped as applied (*N* = 12) with sustainable FDI works are the ones lacking the words policy or reforms in their title but make use of it in their abstract. On the other side, inferential articles lack the word policy in both parts but have still indirectly defended policy-related arguments at later stages. For example, the article of Asongu and Odhiambo [7] implied policy consequences when the authors claimed that: "…First, enhancing trade openness has a net positive impact on CO2 emissions, while increasing FDI has a net negative impact" [7], p. 227. It is assumed that "enhancing trade policy" is a clear inferential policy implication. (*N* = 17) articles inferring policy implications were identified.

#### Journals' perspectives on sustainable FDI policy and implications

Most journals (*N* = 25) published a single article with explicit or implicit content about sustainable FDI policy and its implications in West and Central Africa. Environmental Science and Pollution Research have the most published in this domain (*N* = 8). They were followed by Science of the Total Environment (*N* = 4), Journal of Cleaner Production (*N* = 4). All remaining journals: Journal of Public Affairs, Technological Forecasting and Social Change, Technological Forecasting and Social Change, Sustainability, Transnational Corporations Review, and International Advances in Economic Research, have limited numbers of publications (*N* = 2).

## Method

### Detecting journals with indications on sustainable FDI policy and implications

**Fig. 1:**
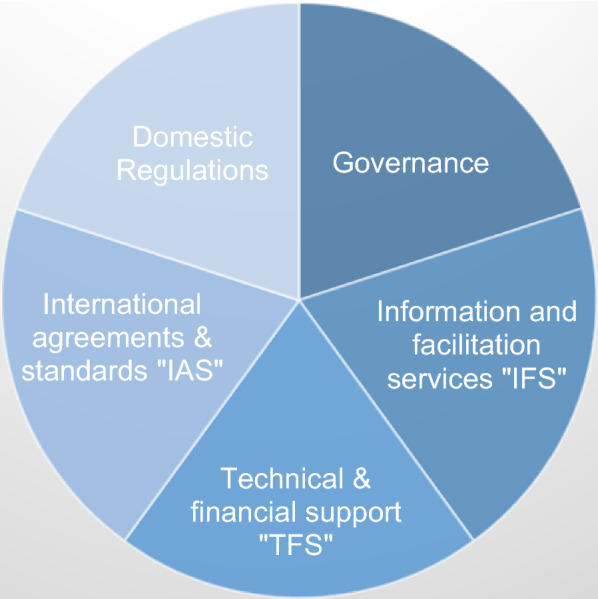
Framework for policies for FDI qualities. *Source*: [36], p. 6

In its conceptual framework named "FDI qualities policy toolkit," the OECD combined the policy framework for investments with other domestic policies to arrive at a framework of policies for FDI qualities [36], p. 6. The framework for FDI policy qualities encompasses five dimensions, as displayed in Fig. 1.

This framework suggests the possibility of measuring sustainability outcomes in the fields of (1) Productivity and innovation; (2) Labor and quality and skills development; (3) Gender equality; and (4) Carbon emissions [36], p. 6. The paper was interested in uncovering which dimensions and fields of the OECD framework were the most applied in West and Central Africa and which journals focus on regional sustainable FDI research. Please note that even if the screened articles may have addressed multiple implications, domains or fields, the research results describe a maximum of three main discussed theoretical or policy inferences within each article.

### Identifying content on policy for FDI qualities

**Fig. 2:**
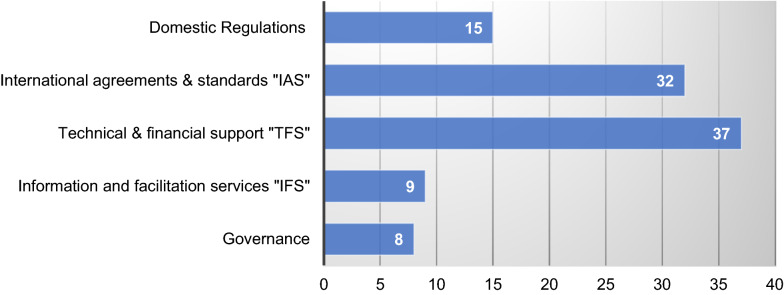
Policies for FDI qualities in Central and West Africa

The classification criteria are based on content analysis. The articles' abstracts, discussions, and recommendations were of particular attention. Therefore, next to the defined parameters, the paper applied the OECD [36] taxonomy criteria for screening all articles. For instance, to satisfy defined internal parameters, the article of Boachie Yiadom and Mensah [10] with the title: "Environmental risk, FDI and tax reforms: why we must worry" was first classified with the type "sustain" and specified as "Obvious." After that, based on the OECD [36] framework, its content was examined to arrive at its classification within the policies dimension of "Domestic Regulation." In their work, the authors argue: "By discomposing tax policy into low and high regimes, we report that countries that deliberately reform tax policy to bait FDI have higher environmental risk" [36], p. 269. These arguments are thought to support a classification of the article as implying domestic policies. Figure 2 presents the number of articles per policy dimension.

### Identifying fields of specific policy implications

**Fig. 3:**
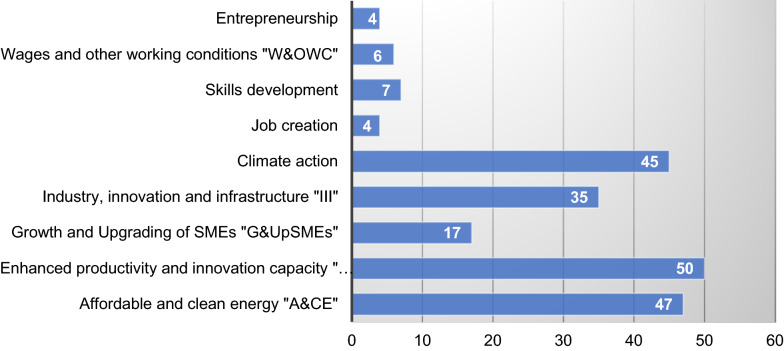
Sustainability outcomes for Central and West Africa

Next to the above classification of the articles to specific policy dimensions, the utilization of the OECD framework enables us to capture the most popular fields and journals focusing on sustainable FDI policy and implication research in West and Central Africa. It is important to note that sustainability characteristics of FDI resumed in the fields of the applied framework are mutually exclusive. Therefore, several fields may be addressed within one publication. For example, Boachie Yiadom and Mensah [10] contended in their article that: "A key consideration from our findings is that reforms in tax policy to lure FDI eventually harm the environment." and recommend "…Second, the existing tax rate or tax laws is not punitive enough to deter FDI from aggravating environmental risk" (2021, p. 282). Following the parameters of the OECD [36], pp. 142–154, these arguments are believed to support a classification of the analyzed field of Productivity & Innovation and Carbon Emission. Most articles investigate FDI sustainability characteristics with regard to Productivity & Innovation- Carbon Emissions (*N* = 53), Productivity & Innovation-Labor & Quality Jobs & Skills Development (*N* = 38), Productivity and Innovation (*N* = 11), and Labor & Quality Jobs & Skills Development & Productivity & Innovation—Carbon Emissions is addressed in a single article (*N* = 1). Deeper implications in sustainability outcomes as provided within the OECD are displayed in Fig. 3. The following dimensions were relevant in the West and Central African literature: Enhanced Productivity and Innovation Capacity "EP&IC" (*N* = 50), Affordable and Clean Energy "A&CE" (*N* = 47), and Climate Action (*N* = 45). “Appendix 2” provides a comprehensive view of  the current classification and related research.

## Results

After the debate on a hypothetical "African growth tragedy" and the perceived pattern of economic growth without economic development [1, 15, 34], the non-resilience of African economies during the first days of the pandemic of COVID 19 revived the need for a discussion about a hypothetical "African sustainable growth tragedy." This review sought to answer whether lawmakers and academia go beyond quantitative macroeconomic rationales to consider more qualitative and sustainable aspects of FDI as defined by the OECD [36] in West and Central. The screening of publications from January 2019 through March 2022 resulted in *N* = 53 (9%) relevant articles for our analysis. Considering that we added a screening factor on regional focus, it is believed that the above percentage is relatively high and therefore implicates that the subject of sustainability is gaining importance in FDI research and policy in West and Central Africa. The review has promising findings about regional applied policies, theoretical domains, and sustainable FDI characteristics. These results and their implications for policymakers, academia, and future research agenda are discussed in the subsequent.

### Perspectives and recommendations for policymakers

Even if policymakers are believed to work in "epistemologically closed systems" [14], p. 498, the fact that they are informed by academia or other sources may help them in getting practical implications within existing policy ecosystems [8], p. 111. This review strived to capture if and which dimensions of sustainable FDI policy frameworks are acknowledged by academia and policymakers in West and Central Africa and contribute to the above objectives. The OECD framework listed many key dimensions (*N* = 4) and sub-dimensions (*N* = 12) for sustainable FDI policy outcomes OECD [36]. The review shows mixed results in policy implications and applied West and Central African research regarding the relevance of some dimensions.

#### On governance and domestic regulations

While Duodu et al. ([17], p. 66101) found that "policies and institutions for environmental sustainability complement with FDI to improve environmental quality in the long run," Appiah-Kubi et al. [5] recommended that: "…. West African nations should endeavor to urge domestic organizations to work ethically through the required foundation of morals and consistence units in associations. … they should guarantee the alleviation of approach impedance in the detailing and usage of corporate regulations" [5], p. 12. These findings highlight the importance of governance, domestic policy, and frameworks for sustainable FDI. The few articles addressing policy and implications of sustainable FDI around domestic regulations and governance may suggest that policymakers do not recognize this dimension well. There is a need for policymakers to engage in this path in the future. In other words, West and Central African policymakers should become more active in tackling challenges related to sustainability dimensions of FDI within their national legal frameworks.

#### On technical and financial support and international agreements and standards

Dauda et al. [13] recognized the need to pursue regional integration and assimilation of innovations into the stages of development of sustainable environmental growth. Technical and financial support coupled with international agreement and standards are key factors in achieving sustainable FDI outcomes. This fact also explains the prominence of technical and financial support policies, international agreements, and standards within the reviewed publications. However, the comparative lack of domestic regulations for sustainable FDI confirms our assumption that the hypothesis of an "African sustainable growth tragedy" can only be addressed through mixed national and international sustainable FDI practices and perspectives. Bekun et al. [9] followed this integrative perspective on policy development and evaluation as they argued that: "… especially to re-engineer the region's economic dynamics if the region must meet the anticipated Sustainable Development Goals 2030" [9], p. 66695. Policymakers are advised to amend national regulations and frameworks to consider international standards in preventing multinationals from exporting economic imbalances that may limit sustainable economic growth and development.

#### On specific outcomes of the OECD framework

The review revealed that not all four main dimensions used by the OCDE framework for measuring outcomes of the policies for FDI quality are on the agenda of policymakers in West and Central Africa. Surprisingly, the dimension of gender equality encompassing employment, wage and non-wage conditions, career orientation, career progression, skill development, and entrepreneurship appears to have not been of interest for regional research about sustainable FDI. Since it is argued that: "FDI can influence gender outcomes through the direct operations of foreign MNEs or indirectly through business linkages and other market interactions with domestic firms" [36], p. 113, policymakers should develop strategies to address the related domestic challenges. Moreover, as sustainable growth cannot be achieved if all genders do not actively participate in the creation of wealth and the realization of green environment projects, it isagreed with Eun Mee ([19] p. 239) that "…education, employment, and the full empowerment of women and girls must be a priority for the SDGs." It is understood that while promoting sustainable FDI, Central and West African policymakers should consider frameworks that particularly strengthen gender equality. In general, the fact that climate-related outcomes are the most inferred shows that policymakers have acknowledged the role that the dimension of carbon emission may play within a sustainable FDI ecosystem. However, as Musah et al. observed: "… improvement in energy efficiency, sustainable infrastructure, and good use of resources (SDG 12) should be promoted by the nations" [31], p. 12313, it is presumed that this process needs to be further pursued and intensified.

Overall, there are additional important aspects that policymakers need to examine critically. For example, policies and implications around the dimensions of growth and upgrading of SMEs (*N* = 12 implications) as well as the ones related to labor & quality jobs & skills development (thereof: job creation "* N* = 3," skills development "* N* = 5"; wages and other working conditions "* N* = 2," and entrepreneurship "* N* = 2") are the least addressed in the literature. This view is also supported by Parente et al. [40] who concluded: "….Our findings indicate that after entry, the MNE sustained its operations in the DRC [Democratic Republic of Congo] by engaging in collective actions and coevolving with key stakeholders within its business ecosystem …Our qualitative data further suggest that the MNE's business ecosystem evolved through three stages-exploring, establishing, and embedding-and that within this ecosystem, the key stakeholders also coevolved with the MNE by adopting new roles over time" [40], p. 275.

It is assumed that Parente et al. [40] emphasize the need to nurture a possible next step of MNE's business ecosystem, which considers dynamic capabilities and workforce development. It is believed that this can be achieved if foreign investors are encouraged to upgrade existing MNEs through policies involving technology transfer, skill development, job creation, and better labor conditions. In addition to the above recommendations, “Appendix 3” provides future policy referential with a detailed view of the related OECD's inferred policies and outcomes that may be subject to particular attention for policymakers.

### Perspective and recommendations for academia

This article was written with the hypothesis that academic research might not be part of futile philosophical games [22], p. 12, but help to understand phenomena and provide policymakers with information on possible practical implications. Since all screened articles addressed, or inferred public policy implications,  and following the reasoning of Adler and Jermier ([22], p. 943), the above premise can be confirmed within the scope of this paper.

#### Tackling the hypothesis of an "African sustainable growth tragedy"

It is noted that subjects around sustainable FDI were less addressed (less than 10%). Additionally, some dimensions of the OECD framework have not been investigated yet in West and Central Africa. For example, it is observed that despite the importance that environmental dimensions gained in theoretical discourses related to sustainable FDI, the same cannot be said about governance, gender equality, social responsibility, or social justice. Perhaps there is a need for academia to question regional perspectives on social justice theories. It is believed that the key to reducing FDI imbalances and promoting sustainable FDI may lie in a policy-related discussion triangulating FDI theories, social justice theories, and labor and diversity theories.

The quality of FDI flows is essential for sustainable economic growth and development [36]. The limited volume of articles addressing non-economic arguments indicates a need for academia to explore this research field. Considering the conclusions of Sichei and Kinyondo [42], it is understood that the inability to attract sustainable FDI in West and Central Africa may result from a combined information asymmetry on risk and negative perception of doing business in the region. Furthermore, the low number of publications related to the dimension of governance (*N* = 8) and the relatively low level of research linked to the dimension of industry, innovation, and infrastructure (*N* = 15) presume a short-term orientation of regional FDI research. It may also indicate a need to explore further how perception, influence, and trust theories may influence the promotion of sustainable FDI in the region.

#### Identifying specialized journals for future research on sustainable FDI

Scholars need guidance for diffusing the results of their research. Following Kamdem's [25] findings, the critical review began without focusing on specific journals. It was argued that identifying specialized journals on sustainable FDI in West and Central Africa may be difficult. While the conclusion of Kamdem [25] cannot be fully confirmed in this research, 579 publications between January 2019 and March 2022, the number of dedicated publications (*N* = 53) reveals the need for specialized publications on sustainable FDI research. The review enables us to identify the leading journals publishing on sustainability issues for FDI and regional economic growth and development. The overview was limited on journals that published more than two articles during the observed period. The list presenting these observations is believed to be of additional value for scholars willing to investigate phenomena around regional sustainable FDI. Table 2 provides this list.

**Table 2:** Specialized journals for future research on regional sustainable FDI

Journals	Number of articles	Cited	SJR	SJR best quartile
Environmental Science and Pollution Research (ESPR)	8	762	0.845	Q2
Journal of Cleaner Production	4	235	1.937	Q1
Science of the Total Environment	4	240	1.795	Q1
International Advances in Economic Research (IAER)	2	96	0.155	Q4
Journal of Chinese Economic and Business Studies	2	82	0.412	Q2
Journal of Public Affairs	2	256	0.221	Q3
Sustainability	2	112	0.612	Q2
Technological Forecasting and Social Change	2	237	2.226	Q1
Transnational Corporations Review (TNCR)	2	106	0.362	Q2

## Research limitations

We focused on research published during the pandemic of the COVID19 and therefore screened research from January 2019 to March 2020. We endeavored to capture the theoretical discourse during the times of pandemics. The focus on articles published in West and Central Africa let us suppose that there is no other research of this kind that may have been achieved. This assumption may be rebutted, especially as conceptual papers or literature review articles were not considered. The small number of research focusing only on the investigated region made us include specific research addressing broader sub-Saharan territories. Critics may argue about a possible defocus. However, as the UNCTAD [45] acknowledged similar patterns within sub-Saharan sustainable FDI arrangements, it can be argued that the outcomes of this research are still of some importance.

As shown in “Appendix 1”, every efforts were made to implement a replicable and transparent methodology and procedures, including the choice of words successively used to screen the data. Based on our understanding, we classified the articles according to the dimensions of the OECD report. Due to the limited number of publications and the regional focus, a similar approach will likely result in the same number of articles. Moreover, the macroperspective of the OECD framework we used to review and classify policy inferences may lead to slightly different results if peers apply a microperspective. In general, utilizing different taxonomies may result in different frequencies in the applied areas of the OECD framework. However, it is believed that despite the above limitations, the research's main premises and conclusions would stand for peer replications and critiques.

## Conclusion

The claim of Kindleberger [26] that imperfect markets are the reason for the observed increase of FDI flows has proven to be particularly relevant in times of economic crisis. This argument has, however, resulted in questioning the spread of economic imbalances, which, in the absence of international frameworks and agreements, sustainable governance and domestic public policy, may, in turn, result in more social injustice and unsustainable economic behavior in the recipient economies. The effort to promote FDI in volume in a context of economic and political fragility or lenient approaches to sustainable policies may have been the reason behind the observed economic growth without development in West and Central Africa. It is argued that if nothing is done, the "African growth tragedy" hypothesis may be intensified and even turn into an "African sustainable growth tragedy."

Under the premise that the quality of FDI is an essential determinant for sustainable growth and may help avoid the above-hypothesized outcomes, the paper questioned which qualitative and specific sustainability dimensions of FDI lawmakers and academia should emphasize to nurture sustainable economic growth and development in West and Central Africa. The conducted review endeavored to capture if such behaviors have been already observed and which policies and implications were significant within this regional economic ecosystem. Based on critical literature review procedures utilizing the OECD framework on policies for FDI qualities, we found that environmental policies and sustainable macroeconomic determinants were on the agenda of academia and policymakers. The screened articles allow, as per date, the identification of domains or determinants with less or even non-addressed policy implications and outcomes. It is observed that there is a theoretical gap in investigating and addressing sustainability outcomes around the subjects of social justice in general and gender diversity in particular. It is recommended that policymakers develop governance and domestic policy frameworks that protect their economies from FDI imbalances and promote sustainable FDI behaviors. Furthermore, academia should, in particular, conduct FDI research linked to sustainable infrastructures, gender quality, social justice, perception, trust, and influence. It is believed that if so applied, such findings may become a catalyst for policymakers and more dedicated policies and implications on sustainable FDI at a regional level.

### Supplementary Information

**Additional file 1. Database extract and screening.**

## Data Availability

Data are provided to the publisher and the collection can be replicated in the mentioned database.

## Appendix 2: Classification of current research

**Table:**

Framework conditions	Related Sustainability outcomes	Articles
Domestic regulations (*N* = 15)	Growth and Upgrading of SMEs	Shittu et al. (2020)
	Enhanced productivity and innovation capacity & Wages, and other working conditions	Martins (2021)
	Enhanced productivity and innovation capacity & Climate action	Yiadom and Mensah (2021)
	Affordable and clean energy	Bekun et al. (2021)
	Enhanced productivity and innovation capacity & Affordable and clean energy	Bekun et al. (2022)
	Enhanced productivity and innovation capacity	Ju et al. (2022)
	Growth and Upgrading of SMEs & Enhanced productivity and innovation capacity	Andoh and Cantah (2020)
	Enhanced productivity and innovation capacity	Andoh and Cantah (2020)
	Growth and Upgrading of SMEs	Orji et al. (2021)
	Industry, innovation and infrastructure; Climate action & Affordable and clean energy	Hess (2021), Gyamfi (2021)
	Enhanced productivity and innovation capacity & Job creation	Mechiche-Alamia et al. (2020)
	Growth and Upgrading of SMEs & Enhanced productivity and innovation capacity	Kodzi Jr. (2021)
	Enhanced productivity and innovation capacity & Entrepreneurship	Ajide (2020)
	Growth and Upgrading of SMEs & Skills development	Osabutey and Jackson (2019)
	Enhanced productivity and innovation capacity & Climate action	Duodo et al. (2021)
	Enhanced productivity and innovation capacity	Ogbuabor et al. (2020)
Governance (*N* = 8)	Enhanced productivity and innovation capacity & Climate action	Asongu et al. (2019)
	Enhanced productivity and innovation capacity, Climate Action & Affordable and clean energy	Adams and Acheampong (2019)
	Growth and Upgrading of SMEs & Wages and other working conditions	Glover (2019)
	Growth and Upgrading of SMEs	Appiah-Kubi et al. (2020)
	Growth and Upgrading of SMEs & Climate action	Wang et al. (2021)
	Industry, innovation and infrastructure; Climate action & Affordable and clean energy	Sarpong and Bein (2020)
	Enhanced productivity and innovation capacity & Climate action	Duodo et al. (2021)
	Enhanced productivity and innovation capacity & Affordable and clean energy	Ogbuabor et al. (2020)
Information & facilitation services "IFS" (*N* = 9)	Industry, innovation and infrastructure; Climate action & Affordable and clean energy	Maconachie (2019), Musah et al. (2020)
	Enhanced productivity and innovation capacity & industry, innovation and infrastructure	Joshua et al. (2021)
	Enhanced productivity and innovation capacity & Skills development	Chen (2020)
	Growth and Upgrading of SMEs & Skills development	Park and Tang (2021)
	Enhanced productivity and innovation capacity & Job creation	You et al. (2020)
	Enhanced productivity and innovation capacity	Nketiah-Amponsah and Sarpong (2020)
	Growth and Upgrading of SMEs	Orji et al. (2021)
	Industry, innovation and infrastructure; Climate action & Affordable and clean energy	Adams and Nsiah (2019)
International agreements & standards "IAS" (*N* = 32)	Enhanced productivity and innovation capacity & Affordable and clean energy	Shittu et al. (2020)
	Enhanced productivity and innovation capacity & Climate action	Martins (2021)
	Affordable and clean energy	Yiadom and Mensah (2021)
	Enhanced productivity and innovation capacity	Bekun et al. (2021)
	Growth and Upgrading of SMEs	Bekun et al. (2022)
	Enhanced productivity and innovation capacity & Wages and other working conditions	Ju et al. (2022)
	Enhanced productivity and innovation capacity & Climate action	Asongu et al. (2019)
	Enhanced productivity and innovation capacity, Climate Action & Affordable and clean energy	Adams and Acheampong (2019)
	Growth and Upgrading of SMEs & Enhanced productivity and innovation capacity	Beer and Loeprick (2020)
	Enhanced productivity and innovation capacity	Fu et al. (2021)
	Enhanced productivity and innovation capacity & industry, innovation and infrastructure	Onyw et al. (2022)
	Growth and Upgrading of SMEs	Osabohien et al. (2021)
	Enhanced productivity and innovation capacity & Affordable and clean energy	Kwakwa et al. (2021), Hongxing, Abban and Boadi (2021)
	Enhanced productivity and innovation capacity & Climate action	Azam and Raza (2022)
	Enhanced productivity and innovation capacity, Climate Action & Affordable and clean energy	Aust et al. (2020), Sarkodie and Strezov (2019)
	Industry, innovation and infrastructure; Climate action & Affordable and clean energy	Gyamfi (2021), Opoku et al. (2021), Musah et al. (2020, 2022), Asongu et al. (2020), Dauda et al. (2021), Mensah et al. (2019), Nathaniel et al. (2020), Tawiah et al. (2021), Acheampong et al. (2019)
	Growth and Upgrading of SMEs & Enhanced productivity and innovation capacity	Andrianarimanana and Yongjian (2021)
	Wages and other working conditions	Ke Rong et al. (2019)
	Enhanced productivity and innovation capacity & industry, innovation and infrastructure	Aloui and Maktouf (2021)
	Enhanced productivity and innovation capacity & Entrepreneurship	Zezethu and Andrew (2018)
	Industry, innovation and infrastructure; Climate action & Affordable and clean energy	Adams and Nsiah (2019)
Technical & financial support "TFS" (*N* = 37)	Enhanced productivity and innovation capacity & industry, innovation and infrastructure	Onyw et al. (2022)
	Growth and Upgrading of SMEs	Osabohien et al. (2021)
	Enhanced productivity and innovation capacity & Affordable and clean energy	Kwakwa et al. (2021), Hongxing et al. (2021)
	Enhanced productivity and innovation capacity & Climate action	Azam and Raza (2022)
	Enhanced productivity and innovation capacity, Climate Action & Affordable and clean energy	Aust et al. (2020), Sarkodie and Strezov (2019)
	Industry, innovation and infrastructure; Climate action & Affordable and clean energy	Gyamfi (2021), Opoku et al. (2021), Musah et al. (2020, 2022), Asongu et al. (2020), Dauda et al. (2021), Mensah et al. (2019), Nathaniel et al. (2020), Tawiah et al. (2021), Acheampong et al. (2019)
	Growth and Upgrading of SMEs & Enhanced productivity and innovation capacity	Andrianarimanana and Yongjian (2021)
	Wages and other working conditions	Ke Rong et al. (2019)
	Skills development	Aloui and Maktouf (2021)
	Enhanced productivity and innovation capacity & Entrepreneurship	Zezethu and Andrew (2018)
	Growth and Upgrading of SMEs & Climate action	Glover (2019)
	Industry, innovation and infrastructure; Climate action & Affordable and clean energy	Appiah-Kubi et al. (2020)
	Growth and Upgrading of SMEs & Wages and other working conditions	Wang et al. (2021)
	Growth and Upgrading of SMEs	Sarpong and Bein (2020)
	Enhanced productivity and innovation capacity & Job creation	Hess (2021), Gyamfi (2021)
	Industry, innovation and infrastructure; Climate action & Affordable and clean energy	Mechiche-Alamia et al. (2020)
	Growth and Upgrading of SMEs & Enhanced productivity and innovation capacity	Kodzi Jr. (2021)
	Enhanced productivity and innovation capacity & Entrepreneurship	Ajide (2020)
	Growth and Upgrading of SMEs & Skills development	Osabutey and Jackson (2019)
	Industry, innovation and infrastructure; Climate action & Affordable and clean energy	Maconachie (2019), Musah et al. (2020)
	Growth and Upgrading of SMEs & Skills development	Joshua et al. (2021)
	Enhanced productivity and innovation capacity & industry, innovation and infrastructure	Chen (2020)
	Enhanced productivity and innovation capacity & Skills development	Park and Tang (2021)
	Enhanced productivity and innovation capacity & Job creation	You et al. (2020)

## Appendix 3: Future policy agenda in Central and West African sustainable FDI research

**Table:**

Policies dimensions in the agenda	Future policy agenda	Current research
Domestic regulations	Growth and Upgrading of SMEs & Enhanced productivity and innovation capacity	Shittu et al. (2020)
	Enhanced productivity and innovation capacity & Affordable and clean energy	Yiadom and Mensah (2021)
	Climate action & Affordable and clean energy	Bekun et al. (2022)
	Enhanced productivity and innovation capacity & Climate action	Bekun et al. (2021)
	Growth and Upgrading of SMEs & Industry, innovation and infrastructure	Andoh and Cantah (2020)
	Growth and Upgrading of SMEs & Enhanced productivity and innovation capacity	Nketiah-Amponsah and Sarpong (2020)
	Enhanced productivity and innovation capacity & Wages, and other working conditions	Kodzi Jr. (2021)
	Industry, innovation and infrastructure & Affordable and clean energy	Hess (2021)
	Entrepreneurship	Ajide (2020)
	Enhanced productivity and innovation capacity	Orji et al. (2021)
	Enhanced productivity and innovation capacity & Employment	Ju et al. (2022)
	Enhanced productivity and innovation capacity, Climate Action & Affordable and clean energy	Gyamfi (2021)
	Enhanced productivity and innovation capacity & industry, innovation and infrastructure	Martins (2021)
	Enhanced productivity and innovation capacity & Career progression, and skill development	Osabutey and Jackson (2019)
	Growth and Upgrading of SMEs & Climate action	Mechiche-Alamia et al. (2020)
Governance	Enhanced productivity and innovation capacity & Climate action	Wang et al. (2021)
	Perception, Trust and Influence Influence	Glover (2019)
	Enhanced productivity and innovation capacity & Affordable and clean energy	Duodo et al. (2021)
	Enhanced productivity and innovation capacity & Climate action	Sarpong and Bein (2020)
	Enhanced productivity and innovation capacity & Skills development	Ogbuabor et al.(2020)
	Enhanced productivity and innovation capacity, Climate Action & Affordable and clean energy	Asongu et al. (2019)
	Enhanced productivity and innovation capacity & Climate action	Adams and Acheampong (2019)
	Growth and Upgrading of SMEs & Skills development	Appiah-Kubi et al. [5]
Information and facilitation services	Enhanced productivity and innovation capacity & Affordable and clean energy	Musah et al. (2020)
	Gender equality	Park and Tang (2021)
	Enhanced productivity and innovation capacity & industry, innovation and infrastructure	Joshua et al. (2021)
	Growth and Upgrading of SMEs	Chen (2020)
	Enhanced productivity and innovation capacity & Affordable and clean energy	Maconachie (2019)
	Enhanced productivity and innovation capacity	You et al. (2020)

## Abbreviations

A&CE: Affordable and clean energy
AfDB: African Development Bank
EP&IC: Enhanced productivity and innovation capacity
FDI: Foreign direct investment
G&UpSMEs: Growth and Upgrading of SMEs
IAS: International agreements & standards
IFS: Information and facilitation services
III: Industry, innovation and infrastructure
IMF: The International Monetary Fund
OECD: Organisation for Economic Co-operation and Development
P&I: Productivity & innovation
P&I: Productivity & innovation
QJ&S: Quality jobs & skills
DRC: Democratic Republic of Congo
SDGs: Sustainable Development Goals
TFS: Technical & financial support
UN: United Nations
UNCTAD: The United Nations Conference on Trade and Development
W&OWC: Wages and other working conditions
WCED: World Commission on Environment and Development
WTTC: World Travel and Tourism Council

## Footnotes

1 *The paper was interested in verifying if the assumption of *Kamdem [25]* about the limited interest of academics in African social phenomena is still valid. It was believed that considering a short period and even during a pandemic would support our endeavor best.*
